# Out-of-pocket payments and catastrophic expenditures due to traffic injuries in Ouagadougou, Burkina Faso

**DOI:** 10.1186/s13561-021-00344-w

**Published:** 2021-12-20

**Authors:** Laurène Petitfour, Emmanuel Bonnet, Isadora Mathevet, Aude Nikiema, Valéry Ridde

**Affiliations:** 1 Heidelberg Institute for Global Health, Heidelberg, 69120 Germany; 2 Institut de Recherche sur le Développement, Bondy, 93140 France; 3 Résiliences, Research Institute for Development, Bondy, 93140 France; 4 OECD, Paris, 75016 France; 5 Institut des Sciences des Sociétés, Ouagadougou, Burkina Faso; 6 CEPED, Research Institute for Development, Paris, 75007 France

**Keywords:** Accidents & injuries, Road traffic injuries, Health economics, Health equity, Health financing, Health insurance, Noncommunicable disease, Trauma care, Catastrophic expenditures

## Abstract

**Objective:**

To estimate the out-of-pocket expenditures linked to Road Traffic Injuries in Ouagadougou, Burkina Faso, as well as the prevalence of catastrophic expenditures among those out-of-pocket payments, and to identify the socio-economic determinants of catastrophic expenditures due to Road Traffic Injuries.

**Methods:**

We surveyed every admission at the only trauma unit of Ouagadougou between January and July 2015 at the time of their admission, 7 days and 30 days later. We estimate a total amount of out-of-pocket expenditures paid by each patient. We considered an expense as catastrophic when it represented 10% at least of the annual global consumption of the patient’s household. We used linear models to determine if socio-economic characteristics were associated to a greater or smaller ratio between out-of-pocket payment and global annual consumption.

**Findings:**

We surveyed 1323 Road injury victims three times (admission, Days 7 and 30). They paid in average 46,547 FCFA (83.64 US dollars) for their care, which represent a catastrophic expenditure for 19% of them. Less than 5% of the sample was covered by a health insurance scheme. Household economic status is found to be the first determinant of catastrophic health expenditure occurrence, exhibiting a significant and negative on the ratio between road injury expenditures and global consumption.

**Conclusion:**

Our findings highlight the importance of developing health insurance schemes to protect poor households from the economic burden of road traffic injuries and improve equity in front of health shocks.

## Introduction

### The economic burden of road traffic injuries in Africa

According to WHO estimates in 2019 [1], deaths and loss of healthy years of life have increased by 50% since 2000. In 2015 the Sustainable Development Goals included the decrease in deaths and injuries due to road traffic (Objective 3.6, [2]). Road Traffic Injuries (RTI) have variable health and economic impacts according to the level of development. Chen et al. (2020) estimate that even though they represented 13.2% of the global population and 14.1% of the disability adjusted life-years in 2015, Sub-Saharan Africa accounts for only 2.1% of the projected economic loss of international GDP between 2015 and 2030, due to its lower level of productivity [3]. However, the burden of road traffic injuries is heavy for low-income countries: it accounts for 5% of Global Domestic Product according to the Global Status Report on Road Safety [4]. Relying on simulations in five middle and low income countries, the World Bank estimated that reducing road mortality and morbidity would increase GDP per capita by between 7 and 22% over 24 years [5].

At a microeconomic level, evidence of RTI impacts in LMIC is growing but still especially thin, partly due to underreporting [6]. Two recent reviews of RTI in Africa and Sub-Saharan Africa respectively include only 15 and 13 countries ([7, 8]). In 2014, Wesson et al. found only 4 studies on African countries for a review of the cost of injury and trauma care in low and middle income countries [9]. They highlight the financial burden caused by RTIs, and advocate for prevention interventions, which are very cost-effective according to most of the papers in their review.

The main concern of the economic consequences of RTI is their potential impoverishing effect, especially if the costs are paid mainly through out-of-pocket expenditures ([10–12]). In addition, the prevalence of catastrophic expenditures among RTI victims is easier to compare between countries than amounts of costs. For instance, a study in Nigeria found that the prevalence of catastrophic out-of-pocket expenditures among their sample of RTI victims was 86% [13]. In Ghana, 45% of injured people requiring surgery in the Komfo Anokye Teaching Hospital faced catastrophic health expenditures [14].

Relying on an original dataset in Burkina Faso, this study aims at estimating the expenditures linked to every admission at the only trauma unit in the capital, estimating the prevalence of catastrophic expenditures due to RTI and analyzing the socio-economic determinants of those catastrophic expenditures.

### Context of the study

Burkina Faso, West Africa, saw its population increase from 11.8 to 19.2 million people between 1999 and 2017, with a concentration in the capital. In the meantime, the number of registered cars was multiplied by 4 between 1999 and 2017, reaching 375,163, while the number of registered two-wheeled vehicles was multiplied by 28 over the same period, reaching 2,329,427 (INSD). Road accidents are frequent but hard to quantify [14, 15]), Burkina Faso being no exception to the underreporting trend in Sub-Saharan Africa. In Ouagadougou, the capital, the population increased from around 709,000 inhabitants in 1996 to almost 2.4 million in 2021. Urbanization is disorganized, leading to wide areas of informal and precarious housing around the city. In 2020 the National Fire Station said it intervened in 12,450 accidents, this number being lower than the total number of accidents for which the police was called ([15–17]). In Ouagadougou, most axes are saturated, causing slow traffic speeds. In this context, if accidents occur, they are expected to cause mostly non-severe injuries [18] so that most of the consequences are economic.

In Burkina Faso in general, a significant part of healthcare costs are paid through out-of-pocket expenditures (35.8% of total health expenditures in 2018 in Burkina Faso, World Bank) and less than 10% of the population is covered by a health insurance plan ([19, 20]). Beogo et al., in 2016 [21], found that out of 1666 people from Ouagadougou who reported an illness or injury in their 2011 survey, 96% paid out-of-pocket. The payment for healthcare at Yalgado hospital’s emergency and trauma department is no exception. For each treatment (kit, analysis, drugs), the patient or the person accompanying the patient must take their prescription to the hospital’s payment-desk and pay before the treatment is administered.

Given the low coverage of health insurance in Burkina Faso and the payment mechanism focused on out-of-pocket expenditures, our main hypotheses tested are the following: (i) the medical prescriptions are determined by the nature of the injuries and are not affected by patients’ socio-economic characteristics (ii) households mostly rely on their own financial resources to cope with the expenses linked to a road accident, and (iii) poor households are the most likely to face a catastrophic expenditure if a RTI occurs.

## Method

### Setting and data population

In 2015, the Yalgado Ouedraogo hospital was the only one in Ouagadougou to provide an emergency trauma unit. Victims of road accidents were brought there directly by both firefighters and ambulances, except if they wanted to be directed to a private health facility. This study encompasses every RTI victim brought to Yalgado Ouedraogo emergency trauma department between January and June 2015.

### Data collection

Data collection includes three questionnaires: a first one filled in by the interns in the trauma unit, on admission of injured patients, describing their lesions and the care provided, and two others administered by means of a phone call to the victim 7 and 30 days after the accident between January and July 2015, concerning socio-economic characteristics, expenses due to the RTI and follow-up of the care required. During the same period, a parallel survey was undertaken in partnership with the Ouagadougou police to register every accident to which the authorities were called, as well as the main information (vehicles involved, victims, location) [15, 16].

Data was collected through SPHINX software, and all analyses were realized with Stata software, v16 (Stata Corp 2013).

### Data analysis

#### Selection of variables

Our out-of-pocket expenditures variable was obtained through the prescriptions obtained by the admissions questionnaire, and the unit prices collected at the Yalgado pharmacy at the time of the survey, and through the patients’ declaration of the prescriptions they left the hospital with, and their possible return to the hospital for healthcare linked to their accident. We know if prescribed care was bought or carried out so as to estimate the extent of healthcare refusal. From those elements we computed our out-of-pocket expenditures variables: all expenses paid for care prescribed at the trauma unit (including the discharge prescription), and expenses linked to the return consult at the trauma unit if there was one. From the Day 7 and Day 30 questionnaires, we also had information on how patients financed their healthcare expenses.

Catastrophic expenditures are usually defined in the literature as representing a certain proportion of a household’s ability to pay. Even though using global household consumption as a denominator has its limits and ideally one should withdraw expenditures linked to basic needs (housing, food) from global consumption level to get the household ability-to pay [22], no disaggregation of the consumption items was available in the questionnaire so that we used the initial measure with global expenditures as denominator ([10, 12]). The 10% threshold was retained, in line with the literature ([13, 23]).

#### Estimation of out-of-pocket expenditures

In a preliminary OLS regression we introduced the expenditure as the dependent variable. The independent variables were characteristic of the injuries: number of lesions, gravity score, location of lesions, and dummies for poly-traumatized patients, programming of a surgery and hospitalization. This step aimed at testing the hypothesis that prescriptions are determined only by the nature of the lesions and not by the patients’ ability to pay.

#### Determinants of catastrophic expenditures

To understand the factors leading to catastrophic expenditures, we estimated an OLS regression model to explain the ratio between healthcare expenditures caused by RTIs in the emergency and trauma department and the total annual consumption of the household. Njagi et al. [24] provide a list of the major determinants of catastrophic expenditures: quintile of wealth, education of the household head, area of residence (rural or urban), presence of health insurance.

## Results

### Profile of the accidents

#### Characteristics of our sample

Among the 1867 patients who were asked to join the study, 1646 accepted (88%). Of those, 1387 could be reached after 7 days (84%) and 1323 after 30 days (80%). Those 1323 patients constitute our study sample. This sample is two thirds male, and young: 31% are under 24 years old, more than two thirds are under 35 years old (see Table 1), and almost all the victims (97%) live in Ouagadougou. 9 victims out of 10 were on two-wheeled vehicles (85% motorcycle, 5% bike).

**Table 1:** Socio-economic characteristics of the sample

	Freq.	Percent
Age categories		
-18	124	9.4
18–24	292	22.14
25–34	492	37.3
35–49	253	19.18
50–64	117	8.87
65	41	3.11
Total	1319	100
Sex		
Female	440	33.26
Male	883	66.74
Total	1323	100
Education level		
None	301	24.43
Primary 1rst year	24	1.95
Primary 2nd year	23	1.87
Primary 3rd year	37	3
Primary 4rth year	131	10.63
Primary 5th year	10	0.81
Primary 6th year	11	0.89
Middle school	248	20.13
Secondary school	236	19.16
University	211	17.13
Total	1232	100
Monthly household consumption		
Less than 30,000 FCFA (57US$)	35	2.84
30,000–79,000 FCFA (57-150US$)	275	22.32
80,000–130,000 FCFA (151-247US$)	115	9.33
130,000–300,000 FCFA (248-569US$)	103	8.36
Over 300,000 FCFA (569US$)	6	0.49
Do not know/Refuse to answer	698	56.63
Total	1232	100
Connected to water network	805	60.85
Connected to electricity grid	801	60.54

With respect to the monthly consumption of their household, 57% of the respondents were not able to choose one of the proposed categories. Among the others (534, 43%), 6.55% spend less than 57US$, 51.5% between 57 US$ and 150US$. A fifth of the subsample spend between 151 US$ and 247US$, and another fifth more than 248 US$. Less than 1% of the subsample spend more than 569US$.

#### Main characteristics of lesions

Most RTI victims of our sample are lightly injured: almost one third of the sample exhibit one lesion only, 60% suffer from 2 lesions at the maximum and less than 2% exhibit more than 6 lesions (see Table 2). 36% of the RTI of the sample are polytraumatized (i.e. wounded at several parts of the body). As a result, categories 1 and 2 of the gravity score (the less serious) represent 66% of the sample.

**Table 2:** Summary statistics about explanatory variables

	Freq.	Percent
Gravity score		
1	373	29.30
2	471	37.00
3	284	22.31
4	135	10.60
5	8	0.63
6	2	0.16
Total	1273	100
Number of lesions		
0	45	3.40
1	394	29.78
2	362	27.36
3	245	18.52
4	142	10.73
5	57	4.31
6	39	2.95
7	20	1.51
8	10	0.76
9	5	0.38
10	2	0.15
37	1	0.08
50	1	0.08
Total	1323	100
Hospitalized	216	16.33
Transferred	216	16.33
Intervention	127	9.60
Polytraumatized	470	35.53
Localization		
Head	131	9.90
Face	321	24.26
Lower members	735	55.56
Upper members	463	35.00
Abdomen	27	2.04
Thorax	33	2.49
Spine	2	0.15
Neck	5	0.38
Open fracture	419	31.67
Closed fracture	127	9.60
Hematoma	74	5.59
Open wound	418	31.59
Superficial lesions	758	57.29

RTI victims are mostly injured at lower members (56%), then upper members (35%) and face (24%). Most common lesions are superficial lesions (60% of the sample) and fractures (40%).

### Analysis of the out-of-pocket expenditures

**Fig. 1:**
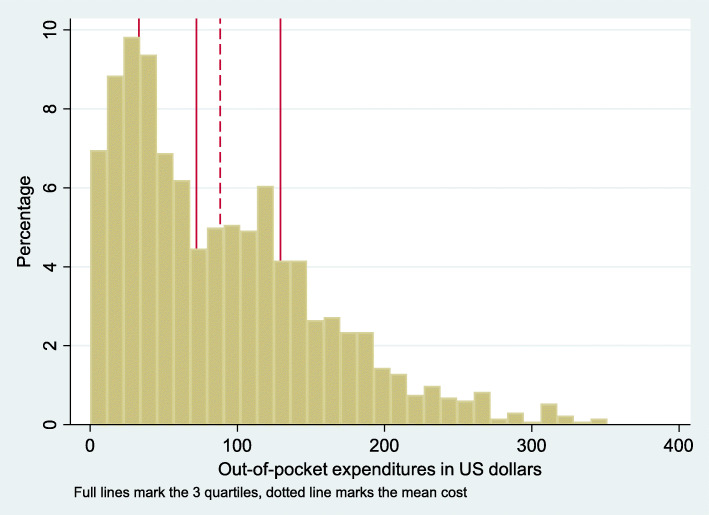
Distribution of the out-of-pocket expenditure

The average out-of-pocket expenditure at the emergency trauma unit is 88 US$, the median 72 US$ (Table 3, Fig. 1).

**Table 3:** Summary statistics for healthcare expenses at the emergency trauma department

	Mean	Min	Max	Std Dev	Q1	Median	Q3	P90	P95	P99	N
**Healthcare expenses (US dollars)**	88,38	0,00	351,25	68,28	33,14	72,15	129,33	183,38	218,88	300,73	1323
**Healthcare expense/ consumption**	0.06	0,00	0.72	0.08	0.02	0.04	0.08	0.13	0.18	0.47	534

Health care refusal is rare (9% for scans and 8% for orthopedic treatments, the most expensive care), even the prescriptions on discharge are widely respected (80% bought all the prescribed items, 20 bought part of them). Patients do not avoid those expenses but find the resources to afford them through different means.

Out-of pockets expenditures are mostly induced by radiographies (30%), then drugs (18%), orthopedic treatments (16.55%) and kits (14%) (see Table 4). The composition of the bill is different between the expense’s quartiles: In the upper quartile (mean value: 165 US$) orthopedic treatments accounts for 40% of the expenses, radiographies for 30%. In the lower quartile (mean value: 17US$), drugs are the first expense (33%) and other expenses are shared between kits (24%), radiographies (15%) and bandages (10%). Orthopedic treatments represent only 3% of the expenses in this quartile. Some prescriptions, like crutches and orthopedic tools, highly increase the bill for the patient.

**Table 4:** Content of Out-of pockets expenditures (%)

	Total
Radio	30.11
Scanner	5.70
Drugs	17.90
Blood exams	1.27
Kits	14.28
Orthopedic treatments (incl. crutches)	16.55
Drugs and bandages at exit	3.70
Consult return	1.40
Other	5.90
Total	100

A month after their accident, more than three quarters of respondents declared that they paid for healthcare thanks to family, 46% used their personal savings, half of the sample used several modes of financing. They contracted a debt in 14% of cases, of an average amount of 123US$, and the proportion of patients who had to borrow money was significantly higher for the fourth quartile of healthcare expenditure. Respondents declared they usually relied on their family (59%) and personal savings (51%) for health expenses.

### Factors determining the healthcare expenditure due to RTI

Variables describing the number, gravity and location of the lesions explain around 49% of the variability of the model. Socio-economic variables (age, education level, activity, consumption level) were introduced in the model, but none was found to be significantly associated with the expense, suggesting that there is no difference in the prescription according to those variables, and validating our healthcare expense variable.

#### Analysis of the ratio between out-of-pocket expenditure and total consumption

The mean proportion between healthcare expenditures due to RTI and annual consumption is 0.06 (see Table 3). Out-of-pocket expenditures represent 10% of the annual household consumption for 19% of the sample.

#### Explanation of the ratio between healthcare expense and total consumption

The most powerful determinants of the ratio between healthcare expense and total consumption are the wealth dummies, all significant with the expected sign. The widest effect is associated to the poorest wealth category, which increases the ratio by 15%. Undergoing surgery or being hospitalized also has a significant and positive impact on the ratio between health expenditures and total consumption (Table 5).

**Table 5:** Determinants of the ratio between the out-of-pocket expenditures due to RTI and annual household consumption

	1	2	3	4	5	6	7	8
VARIABLES	A^a^	B^b^	C^c^	D^d^	A	B	C	D
Had surgery	0.0405**	0.0969***	0.119***	0.00715	0.0515***	0.108***	0.132***	0.0281
	(0.0195)	(0.0281)	(0.0305)	(0.0475)	(0.0153)	(0.0254)	(0.0262)	(0.0447)
Hospitalized	0.0302***	0.0412***	0.0483***	0.154***	0.0313***	0.0422***	0.0495***	0.160***
	(0.00805)	(0.00971)	(0.0102)	(0.0430)	(0.00628)	(0.00767)	(0.00788)	(0.0435)
Household head	0.0108	0.00435	0.0104	0.0197	0.00642	−0.000158	0.00527	0.0154
	(0.00921)	(0.0106)	(0.0109)	(0.0183)	(0.00719)	(0.00847)	(0.00854)	(0.0174)
Gender	−0.00927	− 0.00986	− 0.0120	− 0.0328	− 0.00149	−0.00168	− 0.00297	−0.0235
	(0.00897)	(0.0107)	(0.0107)	(0.0281)	(0.00697)	(0.00865)	(0.00852)	(0.0277)
Age over 50	−0.0162	−0.0250	−0.0262	0.0139	0.00724	−0.000126	0.000831	0.0414
	(0.0227)	(0.0249)	(0.0254)	(0.0437)	(0.0171)	(0.0204)	(0.0196)	(0.0379)
Age under 18	−0.0244***	−0.0301***	−0.0275***	− 0.0137	−0.00592	− 0.0105	−0.00607	0.0105
	(0.00859)	(0.00963)	(0.0100)	(0.0249)	(0.00709)	(0.00830)	(0.00832)	(0.0239)
Age 35/49	−0.0129	−0.00936	−0.0105	0.0105	0.00178	0.00607	0.00663	0.0312
	(0.00807)	(0.00891)	(0.00960)	(0.0222)	(0.00617)	(0.00739)	(0.00755)	(0.0207)
Age 50/64	−0.0142	−0.0141	−0.0168	− 0.0206	−0.00310	− 0.00237	−0.00396	− 0.00950
	(0.0153)	(0.0177)	(0.0189)	(0.0330)	(0.0130)	(0.0157)	(0.0163)	(0.0311)
Water network	0.00472	0.00232	0.00481	0.0118	0.0146	0.0127	0.0162	0.0277*
	(0.0117)	(0.0116)	(0.0144)	(0.0151)	(0.00929)	(0.00906)	(0.0118)	(0.0147)
Electricity grid	−0.0287**	−0.0297**	−0.0330**	0.0170	−0.0157*	−0.0163*	− 0.0180*	0.0343*
	(0.0117)	(0.0117)	(0.0136)	(0.0188)	(0.00838)	(0.00840)	(0.00977)	(0.0188)
Consumption 80,000/130,000					−0.0289***	−0.0302***	−0.0320***	− 0.0688**
					(0.00434)	(0.00573)	(0.00564)	(0.0283)
Consump. Less than 30,000					0.154***	0.166***	0.180***	0.135***
					(0.0321)	(0.0353)	(0.0386)	(0.0378)
Consump. More than 130,000					−0.0452***	−0.0435***	−0.0521***	−0.109***
					(0.00409)	(0.00584)	(0.00523)	(0.0337)
Primary school	−0.0242*	−0.0323**	−0.0359**	0.00951	−0.0103	− 0.0175	− 0.0196	0.0220
	(0.0136)	(0.0150)	(0.0163)	(0.0197)	(0.0107)	(0.0122)	(0.0127)	(0.0176)
High school level	−0.0271*	−0.0266*	−0.0354**	− 0.000716	−0.00540	− 0.00374	−0.0101	0.0246
	(0.0141)	(0.0159)	(0.0169)	(0.0231)	(0.0108)	(0.0129)	(0.0129)	(0.0210)
College level	−0.0416***	−0.0466***	− 0.0510***	0.0243	− 0.0154	−0.0195	− 0.0207	0.0619*
	(0.0150)	(0.0163)	(0.0180)	(0.0326)	(0.0117)	(0.0133)	(0.0140)	(0.0372)
Middle school level	−0.0177	−0.0177	−0.0214	0.0524*	0.000189	0.00135	−0.000673	0.0721***
	(0.0158)	(0.0186)	(0.0198)	(0.0288)	(0.0127)	(0.0158)	(0.0163)	(0.0268)
Constant	0.0964***	0.104***	0.108***	0.0431	0.0599***	0.0647***	0.0648***	0.0157
	(0.0136)	(0.0151)	(0.0158)	(0.0269)	(0.00848)	(0.00994)	(0.00997)	(0.0284)
Observations	533	533	533	480	533	533	533	480
R-squared	0.140	0.194	0.235	0.098	0.419	0.416	0.467	0.152

Robust standard errors in parentheses *** *p* < 0.01, ** *p* < 0.05, * *p* < 0.1

^a^A = Computed emergency cost

^b^B = Computed emergency cost + declared surgery and hospitalization cost

^c^C = Compeuted emergency cost + declared surgery and hospitalization costs or mean surgery and hospitalization costs if the patient declares she/he had surgery or hospitalization but did not give their cost

^d^D = Cost declared by the patients

### Robustness checks

We first investigated whether the chosen method of measurement of out-of-pocket expenditures at trauma unit affected our results by running the same analysis with expenses declared by patients at D7 and D30. The findings remain the same.

Then, as surgery and hospitalization expenses have been highlighted in the literature as the major part of the financial burden of RTI ([13, 25]), we tested whether adding them to the out-of-pocket expenses at the trauma unit would change our results. We do have some information about hospitalization and surgery expenses through the expenditures declared at D7 and D30, though the number of missing information is extremely high. We added declared surgery and hospitalization expenses to the out-of-pocket expenditures at the trauma unit to test the robustness of our results. To cope with the missing values for hospitalization and surgery expenses, we attributed the mean value of non-missing answers for each observation where respondents declared they had surgery but could not say how much they spent for it. We proceeded the same way with the mean declared expenses of hospitalization when respondents declared they were hospitalized but could not give the associated expense. Then we implemented the same procedures as with our initial measure to compare their results.

Results are presented in Tables 6 and 7. The inclusion of the expenses related to surgery and hospitalization only changes the values of the fourth quartile of expenses (25th, 50th and 75th percentiles are close in measure A, B and C) and increases the proportion of observations for which the expenses represent more than 10% than the global household expenditures from 18.9 to 20.4%. It mostly increases the evaluated expenses of observations already in the highest quartile, which is confirmed by an extremely high Spearman rank correlation coefficients between expenses A, B and C (> 0.97, see Table 8). The ranking of observations is different with measures D and E (the correlation coefficient is not significantly different from 0 between Cost E, and A and C (see Table 9). Between the ratios of healthcare to consumption, correlation coefficients are all significant and positive, as the Spearman rank coefficients (see Table 8).

**Table 6:** Distribution of the expenses according to different calculations

Espenses estimations (US$)	mean	P25	P50	P75	P90	P95	P99
A Mean TE service admission	80,74	32,14	68,07	118,91	164,78	191,37	253,06
B Mean TE service admission + declared surgery and hospitalization expenses	88,81	33,09	70,02	121,71	176,29	207,49	280,36
C Mean TE service admission + declared surgery and hospitalization expenses + mean surgery and hospitalization expenses if unanswered	93,16	34,76	73,29	127,40	191,37	239,26	345,82
D Total expenses declared	166,92	33,42	79,08	184,18	348,05	569,64	1708,91
E Sum of declared expenses	165,51	0,95	26,79	87,34	191,78	354,32	1693,72

**Table 7:** Distribution of the ratio between the RTI expenditures and the household annual expenditures according to different calculations of the expenses

variable	mean	p10	p25	p50	p75	p90	p95	p99	Prop. > 0.10	Prop. > 0.25
With Exp. A	0.056	0.007	0.016	0.033	0.073	0.121	0.158	0.446	0.189	0.028
With Exp.B	0.063	0.008	0.017	0.036	0.080	0.136	0.176	0.543	0.204	0.037
With Exp.C	0.066	0.008	0.017	0.037	0.085	0.141	0.205	0.543	0.210	0.044
With Exp.D	0.110	0.020	0.020	0.048	0.116	0.246	0.356	1.032	0.283	0.092
With Exp.E	0.071	0.005	0.005	0.023	0.063	0.140	0.267	1.124	0.161	0.051

**Table 8:** Spearman rank coefficients between costs measures (*if *p* < 0.05)

Spearman	Cost A	Cost B	Cost C	Cost D	Cost E	Ratio A	Ratio B	Ratio C	Ratio D	Ratio E
Cost A	1									
Cost B	0.9841*	1								
Cost C	0.9710*	0.9878*	1							
Cost D	0.5176*	0.5442*	0.5632*	1						
Cost E	0.3824*	0.4164*	0.4180*	0.6453*	1					
Ratio A	0.7752*	0.7525*	0.7370*	0.3120*	0.2467*	1				
Ratio B	0.7786*	0.7843*	0.7699*	0.3501*	0.2910*	0.9800*	1			
Ratio C	0.7767*	0.7813*	0.7872*	0.3680*	0.2913*	0.9726*	0.9888*	1		
Ratio D	0.4142*	0.4337*	0.4483*	0.8397*	0.5433*	0.5357*	0.5606*	0.5737*		
Ratio E	0.3319*	0.3601*	0.3608*	0.5349*	0.9099*	0.4197*	0.4583*	0.4568*	0.6406*

**Table 9:** correlation coefficients between costs measures (*if *p* < 0.05)

coeff corr	Cost A	Cost B	Cost C	Cost D	Cost E	Ratio A	Ratio B	Ratio C	Ratio D	Ratio E
Cost A	1									
Cost B	0.6126*	1								
Cost C	0.8168*	0.7436*	1							
Cost D	0.2551*	0.4392*	0.3288*	1						
Cost E	0.0377	0.0888*	0.0537	0.1244*	1					
Ratio A	0.5519*	0.2269*	0.3834*	0.0162	−0.0207	1				
Ratio B	0.5201*	0.5518*	0.5737*	0.1705*	0.1297*	0.8844*	1			
Ratio C	0.5234*	0.3518*	0.5822*	0.0758	0.0359	0.9160*	0.9484*	1		
Ratio D	0.1523*	0.2006*	0.1834*	0.8224*	0.2665*	0.2256*	0.2736*	0.2472*	1	
Ratio E	0.1518*	0.3884*	0.2882*	0.6555*	0.7607*	0.1535*	0.3428*	0.2714*	0.5525*	1

Once introduced in the econometric model, there is no difference in the significance and sign of coefficients, strengthening the results of our model (see Table 5). We also tested different specifications from the explanation of the ratio between out-of-pockets expenditures and consumption: we used binary variables =1 if the ratio is higher than 0.10, and 0.25, and estimated linear and binary models. Despite the differences induced by the different measures, our main results remain stable: the strongest determinant of the probability of facing catastrophic expenditures is wealth, and the fact of being hospitalized after the accident.

## Discussion

This paper brings empirical evidence of the healthcare cost of RTI, and the extent to which it can represent a catastrophic expenditure for the household of the victims. The mean admission expenditure is 88,38 US dollars. This expense represents more than 10% of the total annual household consumption (a catastrophic expenditure) for 19% of the sample and is paid mainly through personal and family resources. From linear model estimations, we found that prescriptions depend only upon the nature of the injuries and that wealth is the most powerful determinant of the occurrence of a catastrophic expenditure, together with being hospitalized and undergoing surgery.

The characteristics of our sample are typical of the road network in Ouagadougou: the proportion of users of two-wheeled vehicles is higher than in most studies (52% of the sample of RTI victims in Nigeria, [13]). In India, motorcycles represented 70% of registered vehicles in 2010 [26]. On the contrary, the majority of males and distribution of age, concentrated on young adults, are quite similar to other studies ([13, 23]).

The mean out-of-pocket expenditure of 88,38 US dollars can be compared to the mean monthly Burkinabe revenue of 58,30 US dollars in 2015(World Bank), and to the minimum monthly wage in the formal sector, 60,76 US dollars, suggesting the financial burden of RTIs. The incidence of 19% of catastrophic expenditures is lower than in the few similar studies in African contexts. In a Nigerian sample, an incidence of 86% of catastrophic expenditures due to RTI is estimated [13]. Several reasons can explain this high prevalence. First, the risk of underestimation of the cost of surgery and hospitalization is higher in our study because victims were surveyed 7 and 30 days after their accident, while in Nigeria they were surveyed just on being discharged from hospital, minimizing the memory bias. Second, the profile of the sample seems different. Information is not provided about the gravity of lesions, but the average length of stay is 30 days (between 4 and 5 h in our sample), and 53% of their sample underwent surgery (7% in our sample), suggesting more severe accidents, consistent with a higher cost of healthcare. In Ghana, the incidence of catastrophic expenditure was estimated at 40% among injured patients who all underwent surgery.

Our estimation of the proportion of CHE due to RTI is higher than the prevalence of catastrophic health expenditures estimated for Burkina Faso, (between 3 and 5%) [27]. This means that without any other health expenditures in the household during the year when the RTI occurred, 19% of our sample were already in the most vulnerable 3%/5% of the population in terms of impoverishment. People who mobilized their savings and family to finance the expenses of a RTI may have no resource left for other health expenditures.

The fact that wealth is the most important determinant of the probability of victims’ facing catastrophic expenditures is consistent with Njagi et al. (2018) [24] that places it in the first position of the potential determinants. This is consistent with the near absence of health insurance plans in Burkina Faso, which leaves the poorest vulnerable when faced with an unexpected out-of-pocket payment. Being insured significantly reduces the probability for the expenses linked to an injury being catastrophic [23]. In Burkina Faso, the importance of socioeconomic status has already been highlighted. ([28, 29]).

Other findings confirm the economic vulnerability facing the financial risk of RTI. Close family as the central point of protection against health shocks has already been shown in Burkina Faso [30], and in Ghana [31]), The proportion of patients who took out a loan is lower than estimated in a review of 15 African countries, finding a higher propensity to borrow money or sell assets to pay for out-of-pocket payments in Burkina Faso than the other countries in the study (more than 50%) [32]. This suggests that RTI expenses prevent households from using their savings or incurring a debt for a productive reason. They leave households even more vulnerable to any other financial shock.

### Limitations of the study

One of the main limitations of this study is linked to the diversity of the expenses linked to a RTI for the household of the victim. We provided an estimation of the out-of-pocket expenditures in the trauma unit, but data is missing regarding other direct healthcare expenses: 16% of victims were transferred to another service, and 16% were hospitalized. In addition, 30 days after the accident, 52% of the victims declared they sought care somewhere other than Yalgado and we do not have information about the cost of this care.

Data is also insufficient concerning the indirect expenses linked to RTI: vehicle repairs, loss of workdays, while it seems that those expenses are substantial relatively to the direct costs. Among the victims who own the vehicle, 58% had to pay to fix it, spending an average amount of 31,000FCFA. More than a third (38%) of the victims damaged another vehicle, and a third of them had to pay for the damage (mean cost 101,02 US dollars). After 7 days, 70% of the victims still could not work, 41% after 30 days. Those results are in line with the literature: in Nigeria, 13.5% of 127 RTI victims declared themselves unable to go back to work, and 19% of those who could go back to work had to stop working for at least a month [31]. In Sudan, the rate of job loss due to RTI is 9.3% [33]. In a study estimating the global economic burden of deaths caused by RTIs in Iran, productivity loss is found to represent almost all (98%) of the global financial burden, the medical expenses accounting for 2% only of the cost [34]. Even if the proportion of productivity loss would not be that important including non-lethal RTIs, lost wages are a significant part of RTI financial burden, both at the individual and at the social level. Unfortunately, data did not allow us to include those aspects of the financial consequences of RTIs because of the high rate of respondents who could not recall the amounts.

## Conclusion

Our findings suggest that it is essential to develop protection schemes against RTI risks, following the worldwide objective of Universal Health Coverage. Health insurance lowers the probability of catastrophic expenditures when an accident occurs [23], and reduces economic inequalities faced with this risk, though it is still marginal in Burkina Faso. Most RTIs in our sample are non-severe but still represent a financial burden for households. Prevention and information campaigns can have a role to play in reducing the number of road injuries by changing road habits, and have been proved to be cost-effective [9]. These campaigns especially concern two-wheeled vehicle users: wearing a full covering of clothing and closed shoes for instance, using headlights, wearing helmets.

## Data Availability

Data is available at https://www.dropbox.com/home/DONNEES%20ACCIDENTS%202015

## Abbreviations

RTI: Road Traffic Injuries
SDC: Sustainable Development Goals
CHE: Catastrophic Health Expenditures
OLS: Ordinary Least Squares
FCFA: Franc Communité Financière en Afrique
UHC: Universal Health Coverage
